# Speech Technology Progress Based on New Machine Learning Paradigm

**DOI:** 10.1155/2019/4368036

**Published:** 2019-06-25

**Authors:** Vlado Delić, Zoran Perić, Milan Sečujski, Nikša Jakovljević, Jelena Nikolić, Dragiša Mišković, Nikola Simić, Siniša Suzić, Tijana Delić

**Affiliations:** ^1^University of Novi Sad, Faculty of Technical Sciences, 21000 Novi Sad, Serbia; ^2^University of Niš, Faculty of Electronic Engineering, 18000 Niš, Serbia

## Abstract

Speech technologies have been developed for decades as a typical signal processing area, while the last decade has brought a huge progress based on new machine learning paradigms. Owing not only to their intrinsic complexity but also to their relation with cognitive sciences, speech technologies are now viewed as a prime example of interdisciplinary knowledge area. This review article on speech signal analysis and processing, corresponding machine learning algorithms, and applied computational intelligence aims to give an insight into several fields, covering speech production and auditory perception, cognitive aspects of speech communication and language understanding, both speech recognition and text-to-speech synthesis in more details, and consequently the main directions in development of spoken dialogue systems. Additionally, the article discusses the concepts and recent advances in speech signal compression, coding, and transmission, including cognitive speech coding. To conclude, the main intention of this article is to highlight recent achievements and challenges based on new machine learning paradigms that, over the last decade, had an immense impact in the field of speech signal processing.

## 1. Introduction

**Figure 1:**
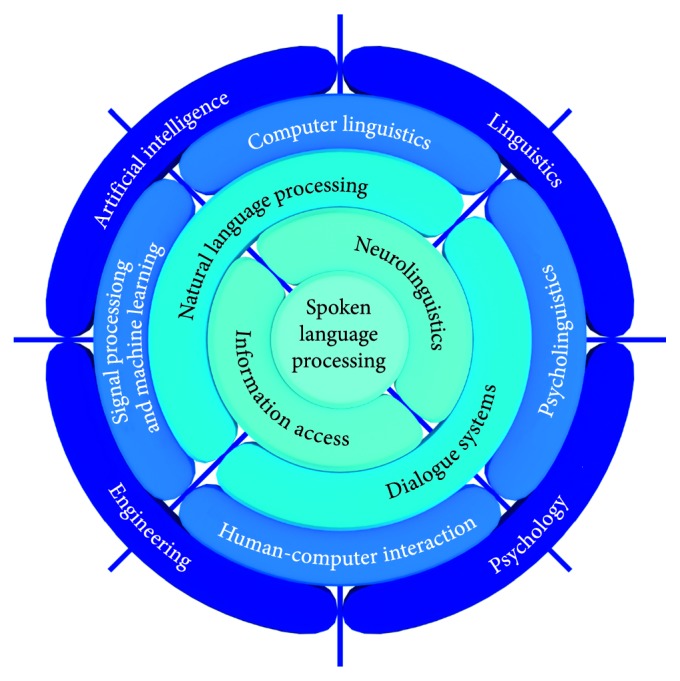
Interdisciplinary nature of speech technologies, i.e., spoken language processing (adopted from [2]).

**Figure 2:**
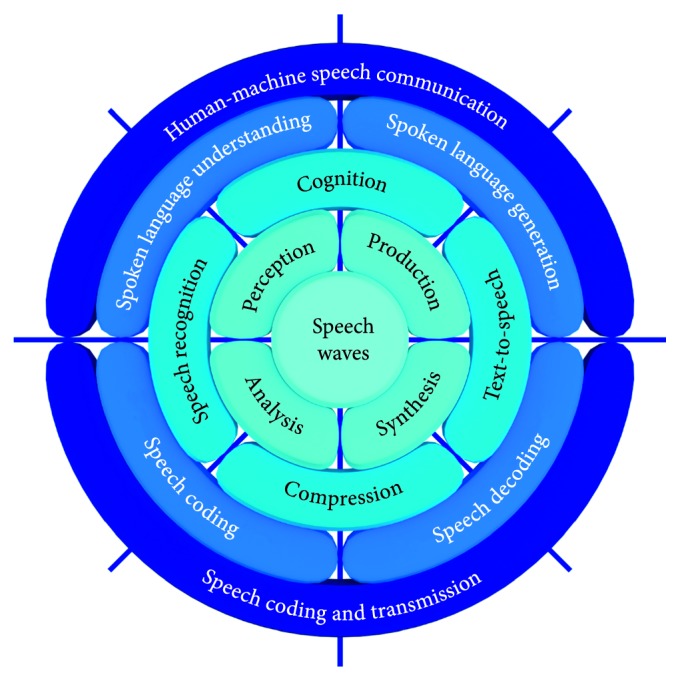
Unified framework that encompasses speech signal processing fields in the scope of the article.

According to Kuhn's theory of scientific revolutions [1], the science makes progress through the revolutionary changes of prevailing scientific paradigms, where a paradigm represents a set of beliefs and values and technical and methodological procedures common to a scientific community. Paradigms define frames and models for solving scientific challenges. New solutions come with new generations who are ready to accept new truths and interdisciplinary approaches. New paradigms appear suddenly and provide new lights to a scientific problem, based on synergy of particular and specialized knowledge consolidated into a functional and coherent unity. Speech technology community investigates spoken language processing as an interdisciplinary research area (Figure 1), [2]. After a short retrospective of the main scientific paradigms based on the knowledge of speech production and auditory perception, this article presents new achievements and perspectives based on the new machine learning paradigm related to neuroscience and advanced signal processing.

The roots of speech signal processing research were closely related to the needs of speech signal digitization. The pioneering solutions were deployed during the World War II due to a need of secure communication between the Allies. The system was named SIGSALY, and it utilized pulse-code modulation (PCM) to enable the first transmission of voice using digital equipment [3]. In the next decades, the focus of researchers was on standardizing rules of digital telephony in order to provide high quality of reconstructed speech signal in the wide range of speech signal variances [4–7]. The compression paradigms regarding these systems have not changed significantly for decades. Particularly, the focus of research has slightly been moved toward improving the signal quality at the receiving end or toward reducing the required bit rate [8–13]. However, the significant development of computer technology in the last decade has enabled research into new approaches to advanced speech signal processing including adaptive machine learning methods [14]. Recent trends include cognitive speech coding so that there is a paradigm shift from perceptual (auditory) toward cognitive (auditory plus cortical) speech signal processing [15].

Modern speech technology systems rely on interdisciplinary research in the areas of multimodal signal processing and artificial intelligence, and a number of methods and algorithms have been developed with the aim of solving various problems: dialogue systems based on speech recognition and synthesis, including emotional speech, speaker identification and verification, as well as speech signal coding and transmission, denoising and detection of signals in the presence of noise, quality enhancement, and medical diagnostics based on the analysis of human voice. Recent progress in most of these speech technology topics will be discussed in more details in the following sections.

Spoken language processing (SLP) is an interdisciplinary research area that has attributes of computational intelligence. SLP lies in the intersection of linguistics, psychology, engineering, and artificial intelligence (AI) [2]. Advanced signal processing and machine learning methods are positioned in the adopted view to the interdisciplinary character of SLP, and both interconnections and intersections of different disciplines are shown and presented in a novel point of view (Figure 1). Instead of using the original term “pattern processing” in Figure 1, we have opted for the more common term “signal processing and machine learning (SP&ML),” which represents the overlap between the community of engineering and AI disciplines. With linguistic aspects included, they compose the natural language processing (NLP) field. Human-computer interaction (HCI) draws experience and methodology from the fields of engineering and psychology, and with the knowledge from linguistics included, they form a basis for the study and development of dialogue systems.

The interconnection of psycholinguistics and AI is the foundation of cognitive science or neurolinguistics. Neurolinguistics has been treated here as the neuroscience of speech. Neurolinguistics is presented in Figure 1 as dominantly linguistics discipline but connected to AI through computer linguistics which is on the intersection between AI and linguistics from one side and also connected to psychology through psycholinguistics, which is on the intersection between psychology and linguistics from other side. Neurolinguistics is on the opposite side from the engineering point of view. The neuroscience of speech can also be considered as an area of cognitive science, and cognition is inherent part of both speech perception (in the phase of understanding, the meaning of the message conveyed by spoken language) and speech production (in the phase of composing, a message intended to convey a certain meaning). Finally, SLP combines knowledge from the interdisciplinary areas of SP&ML, HCI, psycholinguistics, and computer linguistics, or more precisely NLP, cognitive sciences, dialogue systems, and information access.

Speech technologies are based on speech signal processing that spans a wide range of topics, while the focus in this review article is on three areas where the authors have the most expertise:

- Fundamental topics (speech analysis and synthesis, sound waves and speech features, speech production, auditory perception, and cognition including the linguistic aspect)
- Dialogue systems based on speech recognition and text-to-speech synthesis (emotional speech recognition and text-to-speech synthesis including voice and style conversion)
- Speech coding, compression, and transmission

Speech technology fields within the scope of the paper are presented in Figure 2 as a unified framework that encompasses covered topics, showing their complementarity, ranges and borders, interconnections, and intersections in the interdisciplinary area of SLP.

A brief retrospective and some perspectives of the speech technology fields shown in Figure 2 are presented in the following sections. Fundamental topics are shown in the middle of Figure 2 and presented in Section 2, covering speech production and perception analysis, including cognitive and linguistic point of views. More details related to the progress in speech recognition and speech synthesis, as well as their contributions to a new generation of human-machine speech dialogue systems, are presented in Section 3. Finally, the progress in speech signal compression, coding, and transmission is presented in Section 4, including contributions of the authors to the area. Most of these advancements are based on the new deep learning paradigm and our better understanding of neuroscience and modelling of cognitive aspects of spoken language communication.

## 2. Progress in Speech Analysis and Knowledge of Spoken Language Nature

Knowledge related to the nature of spoken language is essential for efficient coding and transmission as well as satisfactory real-time human-machine speech interaction. Speech models based on either speech production or auditory perception were inherent parts of most successful algorithms. Most recent neuro-inspired computational models are based on knowledge of cognitive speech processing models [16]. After a brief review of sound pressure waves and speech signal features, speech production and auditory perception including cognitive and linguistic points of view will be elaborated in more detail in the following subsections.

### 2.1. Sound Pressure Waves and Speech Signal Features

Sound propagates as a continuum of acoustic waves (sound pressure), and, once received, it can be recorded, digitized, coded, transmitted, processed, and reproduced. In case of speech sounds, frequencies relevant for recognizing what was said and who has said it are located mostly below 4 kHz and hardly ever above 7-8 kHz, which is just a portion of the entire frequency range of the human auditory sense [17]. This fact was the basis of the design of analogue telephone communication systems, including the choice of microphones used. For that reason, a speech signal is sampled at 8 kHz (for a basic level of quality) or 16 kHz (if a higher level of quality is desired). It is also well known that the dynamic range from the softest to the loudest sounds in average human speech is approximately 40 dB. Even if whisper and elevated voice are included, this dynamic range is rarely above 50 dB [14]. For these reasons, the requirements for a microphone needed to record voice are typically less strict than in case of recording, e.g., music. As to quantization, it is known that each bit contributes to the signal-to-noise ratio (SNR) by 6 dB, which means that the quantization noise is practically inaudible if 8 bits are used for coding every sound sample [4]. Thus, the typical case of using the sampling rate of 8 kHz and 8 bits per sample produces the bit rate of 64 kbits/s. A lot of effort has been invested to reduce this rate without significant loss of quality at the receiving side, and Section 4 is devoted to this subject.

Digitalization including quantization is the basis of all digital speech processing techniques. If the aim is to represent speech compactly and robustly, as is the case in automatic speech recognition or most types of speech coding for transmission, one of the basic questions is the selection of relevant features that will enable fast, accurate, and robust recognition of speech (or the speaker, language, or even emotion), and/or fast and efficient speech coding for transmission without significant loss of quality. Linear predictive coding (LPC) and LPC analysis have fundamental significance in speech signal modelling and speech feature estimation [18]. Many speech coding schemes are based on LPC including Low Delay-Code Excited Linear Prediction (LD-CELP) coding scheme defined by G.728 standard, Conjugate Structure Algebraic Code-Excited Linear Prediction (CS-ACELP) coding scheme defined by G.729A standard, Algebraic Code-Excited Linear Prediction coding scheme defined by G.723.1 standard, and Adaptive Multi-Rate Wide-Band (AMR-WB) coding scheme defined by G.722.2, standards which are used in today mobile voice communication and VoIP [5, 7].

One of speech production models is also based on LPC analysis and provides speech feature sets describing speech spectrum, which is most important for speech recognition [19]. The main scope of speech signal and data processing in real time (or limited time) is to reduce the amount of data (speech features), while providing high quality of representation of such a reduced signal, that is, data source. The realization of this goal is supported by statistical signal and data processing as well as methods and algorithms which deal with signal and data reduction [20]. The most efficient methods and algorithms incorporate adaptation, and these topics will be elaborated in more details in the next sections.

### 2.2. Speech Production and Auditory Perception

**Figure 3:**
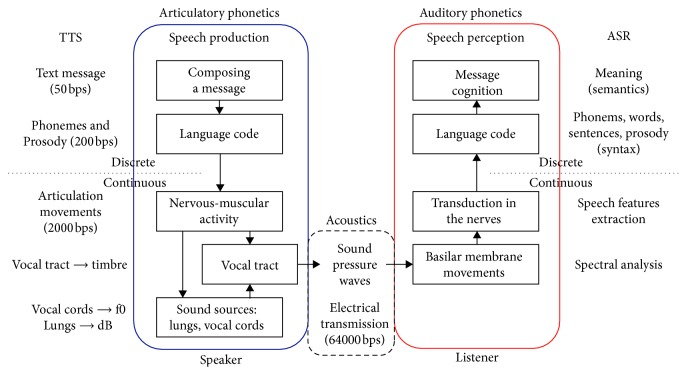
Block diagram of speech production and speech perception and corresponding processes performed by machines carrying out text-to-speech synthesis (TTS) and automatic speech recognition (ASR).

Figure 3 shows a block diagram of both speech production and perception. Text-to-speech synthesis (TTS) and automatic speech recognition (ASR) are shown in parallel as corresponding processes performed by machines. Speech and language are learned, while the sense of hearing is innate. There are a lot of differences among human and machine speech production and perception, but the increase in the ability of machine learning paradigms to simulate human speech production mechanism, as well as auditory perception and cognition abilities, will inevitably bring about an increase in the accuracy of ASR and naturalness of TTS.

Speech communication between humans begins and ends at the cognitive level of message composition and interpretation. Taking into account the average speech rate of 10–12 phones per seconds and the number of phones in a language, which typically corresponds to 5 or 6 bits needed to encode them, a speech message conveyed as text could be considered to correspond to a bit rate of 50–60 bits per second. The speaker plans not only what to say but also how to say it—(s)he controls the volume, speech rate, and intonation (prosody)—any of which can carry linguistic, and also paralinguistic and extralinguistic information [21]. With that information added, the bit rate can be considered to increase to several hundreds of bits per second.

Once the speaker decides what to say and how to say it, an appropriate sound wave is produced through nervous and muscular activity [22, 23]. In that, phones are not pronounced in isolation, but the articulatory targets required for corresponding phonemes are rarely reached, leading to the coarticulation effect, which aggravates the task of ASR. Most often, the entire speech apparatus is considered through the source-filter model, where the activity of vocal folds defines the excitation and the remainder of vocal tract acts as a filter and shapes the sound spectrum [19, 21]. Besides being dependent on the phone, the acoustic features of the speech signal at a particular moment also carry information relevant to the speaker and thus represent a biometrical feature which can reveal the speaker's identity [24] and possibly other factors related to the speaker or to the message. Including the influence of speaker variability, the bit rate at this level increases to several thousand bits per second. This segment of speech communication is studied by articulatory and acoustical phonetics, and its machine counterpart is TTS, namely, the module charged with the production of the artificial speech signal itself.

Distribution of speech sample amplitudes is nonuniform, and this knowledge is used in nonuniform speech signal coding defined by *µ*-law and A-law [25], while some new research results provide better solutions based on adaptive algorithms. The speech production mechanism articulates a series of phonemes nonuniformly, according to an empirical statistical law formulated by George Kingsley Zipf, a linguist [26], referring to the principle of the least effort from evolutionary biology field: interlocutors try to understand each other using phonemes and words that are easier for production and perception in a particular context. The knowledge of phoneme and word statistics has been introduced into ASR algorithms long ago, and stochastic speech models like Hidden Markov model (HMM) [27] were the prevailing scientific paradigm and represented the state of the art in speech recognition and synthesis community for decades.

On the other side, the continuum of acoustic waves reaches the ear of the listener and certain frequencies excite the eardrum, and over the malleus, incus, and stapes, they excite the cochlea, where spectral analysis is performed, based on the movement of the basilar membrane, whose length is about 35 mm [17, 22, 23, 25, 28]. The hair cells in the cochlea respond to different sounds based on their frequency so that high-pitched sounds stimulate the hair cells in the lower part of the cochlea, while low-pitched sounds stimulate the upper part of the cochlea [28]. Thus formed neural impulses are sent to the central auditory system in the brain [22], and based on spectral differences, the brain recognizes relevant acoustic differences and attempts to recover the string of phones that the original message was composed of, taking into account its language model (at the level of morphology, syntax, semantics, and pragmatics). It can thus be considered that the task of ASR is to reduce the bit rate of, e.g., 64 kbits/s (digitized speech) to a bit rate of 50–60 bits/s (plain text), which would correspond to the textual contents of the message without speech prosody.

However, speech perception, which principally relies on the sense of hearing, is a nonlinear process. As is the case with other human senses (vision, taste, touch, and smell), auditory perception of both sound pressure level (SPL) and fundamental frequency (f0, pitch) follows the Weber–Fechner law [28] from psychophysics: a change perceived as linear corresponds to an exponential change in the physical stimulus. Apart from SPL and pitch, perception of sound is affected by the distribution of sound energy across frequencies, i.e., the spectrum of the sound, which usually represents a mixture of a sequence of discrete frequency components (timbre), as in the case of periodic sounds, and a continuous mix of nonharmonic or random frequency components, as in the case of various types of noise [22, 28]. This is why common speech features like cepstral coefficients are considered to be located at frequencies rescaled from Hz to mel-scale–MFCC; they are estimated by cepstral analysis from speech frames of 20–30 ms together with their first and second derivatives calculated from several successive frames [29].

Auditory scene analysis is the process by which the auditory system separates individual sounds in natural-world situations [30, 31]. Regardless of whether sound is received by a human ear or a microphone, the incident sound pressure wave represents a sum of pressure waves coming from different individual sources, which can be either human voices or any other sound sources. These sounds usually overlap in both time and frequency. Nevertheless, the human auditory system is usually able to concentrate on an individual sound source at a time [23, 31]. While listening and separating one source, the listener constructs a separate mental description for that source. Although he/she cannot actively listen to two sound sources simultaneously, he/she can switch immediately his/her attention from one to the other [30]. For example, if a student listens to the teacher, he ignores the noise from LCD projector and a colleague who may be speaking to him; if he switches the focus to his colleague, he cannot actively listen to the teacher anymore. Furthermore, if a human listener follows the context, he/she is able to reconstruct some phonemes or entire words that he/she may not be able to hear for some reason. Humans are as successful in sound separation as they are more experienced in real-word situations and they always analyse the incoming signal using heuristic processes. As the ultimate step of the hearing process, human auditory cortex constructs a cognitive representation of the received sound wave. Without the cognition step, sound waves coming to the ears are not perceived. Heuristic analysis is based on (ir)regularities in the sum of underlying sounds.

Individual sounds differentiate from each other in at least one of the following dimensions: time, space, and frequency spectrum [28, 31]. Temporal and spatial sensations in the human auditory system are presented in more details in [32]. In the time dimension, two sounds can have some onset/offset asynchrony. In a specific environment, binaural hearing enables the localization of sound sources, which is easier, but also often more important, in the horizontal plane where human ears are positioned than in the vertical plane. The spectrum of frequency components can determine the perceived pitch, timbre, loudness, and the difference in the spectra of sounds received by both ears enables the localization of sound sources [23, 31, 32]. Pitch is related to the fundamental frequency f0 in periodic sound waves such as musical tones or vowels in speech; their spectrum consists of f0 and its harmonics. Temporal variation of f0 results in melody in music and intonation in speech. Timbre represents a specific distribution in the intensities of f0s and its harmonics in the spectrum. Two renditions of the same tone from two different musical instruments, having the same f0, will have different timbres due to the difference in the relative intensities of particular harmonics (the spectral envelope), and as a result, they will sound different [22]. If a sound spectrum does not contain just harmonic tones (f0s and their harmonics), the spectrum is not discrete; sound spectrum is rich with frequency components in parts or in the entire frequency range of the human auditory sense. Such sounds, with a spectrum that is more or less continuous, are much more frequent in nature (e.g., noise of a car or a machine or any transient noise). Magnitudes of spectral components contribute to the loudness; sound pressure level is defined in dB relative to the threshold of hearing at 1 kHz (20 *µ*Pa) and has range 0–120 dB to the threshold of pain [17, 22]. To conclude, two sounds can be separated from each other in an auditory scene analysis according to the differences in loudness, pitch (f0, if present), and timbre or spectrum as a whole, as well as in their temporal and/or spatial variations that can create a variety of sound impressions.

Acoustic signals are received by a listener and transformed into linguistic and nonlinguistic categories, but it is not known exactly how. There is ongoing research on neurophysiology of speech communication using the latest advances in invasive and noninvasive human recording techniques, with the aim to uncover fundamental characteristics of cortical speech processing [16]. The research team in question has studied phonetic feature encoding and mechanisms of noise robust representation of speech in auditory cortex based on the evidence that humans and animals can reliably perceive behaviourally relevant sounds in noisy and reverberant environments.

Neuro-inspired computational models try to provide progress in artificial deep neural network (DNN) performance, based on better understanding of the representation and transformation performed by these models. A case study in ASR given in [33] attempts to identify the mechanisms that normalize the natural variability of speech and compares these mechanisms with findings of speech representation in the human auditory cortex. The aim is to compare DNNs with their biological counterparts, identify their limitations, and reduce the performance gap between biological systems and artificial computing. For example, a human is able to concentrate on one speaker voice and ignore other sounds and voices [23, 31], based on their differences in spatial positions, pitch, and timbre, coherence of changes in level and/or frequency, and time characteristics (onset/offset asynchrony) [30]. An algorithm aimed at focusing on one speaker in a group of many speakers based on deep attractor network is proposed in [34], based on similar principles. It has been shown that switching attention to a new speaker instantly changes the neural representation of sound in the brain. An adaptive system should change the sensory representation in real time to implement novel, task-driven computations that facilitate the extraction of relevant acoustic parameters.

Human listeners have a remarkable ability to understand quickly and efficiently the world around them based on behaviour of known sound sources. Moreover, they are able to pay attention and focus on the meaning of speech of a particular speaker. Attentional focus can be integrated into HCI dialogue strategy [35], while data related to human cognitive effort can be used in postprocessing and improvement of the performance of ASR systems [36]. Humans are able not only to separate one speaker or concentrate only to one sound source but also to group more sound sources and hear, e.g., the entire orchestra as one musical sound based on harmonicity and synchrony of particular sound sources. Concurrent and sequential grouping processes are described in more details in [37].

The role of the nonlinearities in DNN in categorization of phonemes by their nonuniform and nonlinear warping of the acoustic space are studied in [38], as well as the way perceptual invariant categories are created. Biological neurons are able to dynamically change the synaptic efficacy in response to variable input conditions. It is called synaptic depression and when it is added to the hidden layers of a DNN trained for phoneme classification, ASR system becomes more robust to noisy conditions without explicitly being trained for them. The results from [39] suggest that more complete neuron models may further reduce the gap between the biological performance and artificial computing, resulting in networks that better generalize to novel signal conditions.

### 2.3. Engineering vs. Linguistic Point of View to NLP as a Typical AI Topic

The mechanism of speech production and the physical component of sound perception are relatively well-studied topics [22, 31], while cognitive aspects of speech communication still represent a widely open research area. All aspects of human-machine speech communication that are related to linguistics, such as natural language processing (NLP), cognitive sciences—neurolinguistics, and dialogue management (see Figure 1), represent great challenges to the scientific community. In the recent past, the development of speech technology and spoken dialogue systems has gained most momentum from the engineering disciplines, through the possibility of automatic learning from vast quantities of data, in terms of development of computational facilities, complex learning algorithms, and sophisticated neural model architectures addressing particular phenomena and problems of cognitive linguistics. At the same time, cognitive speech sciences mostly remain outside of the scope of the immediate interest of engineering disciplines relevant to speech technology development. Nevertheless, the knowledge in these areas overlaps in the concept and scope with machine learning, which, inspired by neurosciences, has brought about progress not only in human-computer interaction and computational linguistics but also in the area of spoken language processing, which lies in their intersection. This is indicated in Figure 1, which also shows a relatively wide gap between cognitive sciences (neuroscience) and psycholinguistics on one side and predominantly engineering disciplines on the other.

As regards the role of machine learning in the development of speech technology, it has offered a powerful alternative to models dependent on linguistic resources and modules performing particular linguistically motivated subtasks. Linguistic resources such as dictionaries and speech databases are typically quite expensive and time-consuming to collect and annotate, while the development of modules that compose a speech technology system requires deep domain knowledge and expert effort. In the last two decades, some of the tasks performed by rule-based systems or simpler machine learning methods have, one by one, been overtaken by neural networks. Namely, in the case of acoustic speech recognition, neural networks have been shown to outperform hidden Markov models (HMMs) in acoustic modelling [40] but have also outperformed classical *N*-gram language models in terms of generalization, using either architectures based on long short-term memory (LSTM) neurons [41] or recurrent neural networks (RNN) [42]. Solutions based on neural networks have been shown to reach human parity in tasks as complex as casual conversational speech recognition [43]. In combination with a range of data-synthesis techniques for obtaining large quantities of varied data for training, it is now possible to obtain an end-to-end ASR capable of outperforming state-of-the-art pipelines in recognizing clear conversational speech as well as noisy one [44, 45]. They have also been used in multimodal speech recognition, i.e., recognition of speech from audio and video [46]. The task of speech synthesis is a more language-dependent one, and in that it is more challenging since it aims to reintroduce the redundancy which is lost when speech is converted into text, and to do it in such a way that, among a multitude of prosodic renditions of a particular utterance, it produces one that the listener will consider acceptable in a given context. Here again, neural networks have shown to overperform classical models working on parameterized speech such as HMMs [47, 48] in acoustic modelling, and they have also been employed for prosody modelling [49] as well as modelling of acoustic trajectories [50]. Neural networks have also addressed the problem of a somewhat muffled character of synthesized speech due to the use of a vocoder, by performing synthesis of raw speech waveforms instead [51]. Finally, to overcome the need for sophisticated speech and language resources that require deep domain expertise, a range of end-to-end architectures were proposed, with the ultimate end that the system should be trained on pairs of text and audio, exploiting the capability of neural networks to automatically develop higher-level abstractions [52]. The flexibility of such a powerful data-driven approach in comparison with classical speech concatenation synthesizers has also brought significant progress in the area of multispeaker TTS and speaker adaptation [53–55] as well the ability to conform to a particular speech style or emotion [56]. This is particularly relevant as it coincides with the emergence of applications such as smart environments, virtual assistants, and intelligent robots, demanding high-quality speech synthesis in different voices and different styles and conveying different emotional states of the perceived speaker [57]. Other language technology tasks have also been successfully addressed by neural networks, such as question answering [58], text classification [59, 60], machine translation [61, 62], and sentiment analysis [63]. Neural networks have also been used as a powerful linguistic tool, for modelling sentence syntax [64] or exploring particular linguistic phenomena such as establishing word representations in vector spaces [65]. However, rather than providing a decomposition of the problem and a clear analytical insight into it, neural networks provide an alternative, data-driven point of view, and thus cannot be considered a classical tool of theoretical linguistics. On the other hand, their performance in solving these problems justly makes neural networks state of the art in the development of speech technology.

## 3. Progress in Speech Recognition and Synthesis, as well as Dialogue Systems

**Figure 4:**
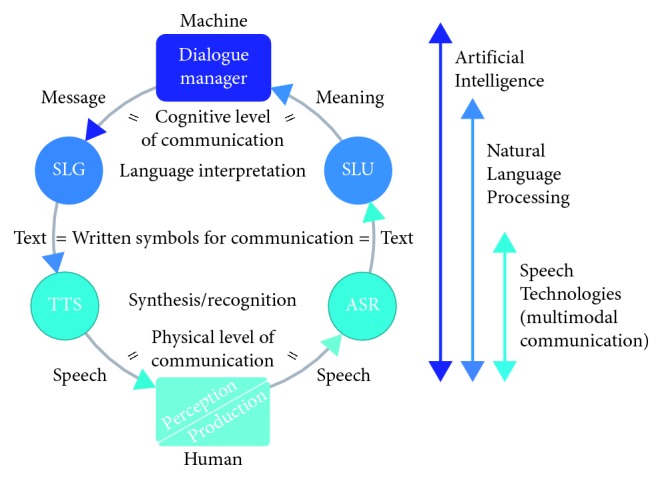
Components of a human-machine speech dialogue system.

Apart from automatic speech recognition (ASR) and text-to-speech synthesis (TTS), a human-machine speech dialogue system also includes a dialogue management module with corresponding dialogue strategies and language technologies for spoken language understanding (SLU) and spoken language generation (SLG), as illustrated in Figure 4.

This section presents some achievements in the field of speech technologies such as ASR and TTS. They have been developed with an effort to combine interdisciplinary knowledge from different areas such as linguistics, acoustics, computer science, and mathematics. Signal processing engineers usually have integrating roles among linguists from one side and mathematicians from the other side.

### 3.1. Progress of Automatic Speech Recognition Systems

Research and development of ASR systems began in the 1950s in Bell Labs, with simple digit recognition systems, and since then the recognition tasks have become more complex—from the recognition of isolated digits, then isolated words, then continuously spoken words in a silent environment, up to the recognition of spontaneous speech in a noisy environment. Consequently, the complexity of the algorithms used also increased drastically. A brief review of historical development of ASR can be found in [66]. There were three important moments in the development of ASR systems: introduction of mel-frequency cepstral coefficients [67], introduction of statistical methods (hidden Markov models (HMM) with Gaussian mixture models (GMM)) [68], and introduction of deep neural networks (DNN) [69]. This development was also supported by the technological development in the computer industry as well as the increase in the amount of data available for training these systems.

The domination of DNNs in ASR started with [40], which showed that feedforward DNN outperforms GMM in the task of estimation of context-dependent HMM state emitting probabilities. For a small database, such as English Broadcast News (about 30 h of training data), the difference in word error rates (WER) was not significant, but for the Switchboard database, which is bigger (about 300 h of training data), the difference became substantial. Further improvement of DNN was based on better optimization, new activation functions, new network architectures, new speech preprocessing methods, and leveraging multiple languages and dialects [70]. One of the important findings was that layer-by-layer pretraining using restricted Boltzmann machines (RBM) is not obligatory and that backpropagation algorithm is sufficient for training in case of a large quantity of available training data as well as a large number of units in the hidden layers. Additionally, LeCun et al. showed in [71] that in case of sufficiently wide DNN (large number of units in a layer), there is no problem with the local minima and that the values of local minima are very close. The next big step was a complete elimination of HMM from the model. Graves and Jaitly in [72] reported a speech recognition system that directly transcribes audio data with text, without requiring an intermediate phonetic representation. The system is based on a combination of the deep bidirectional long-short term memory (LSTM) recurrent neural network architecture and the connectionist temporal classification (CTC) objective function. Such a direct mapping of an audio signal into a grapheme sequence allows easy application of the system on new languages such as Serbian [73]. Inspired by CTC, Povey at al. in [74] developed lattice-free maximum mutual information using phone *n*-gram language model starting from randomly initialized neural networks. This method was also successfully applied to Serbian [75]; i.e., the relative reduction of WER was about 25% with respect to the best previous system.

### 3.2. Progress of Speech Emotion Recognition

Since humans are not always rational and logical beings—emotions play very important aspects in acceptance of new products and technologies [76]. The earliest attempts to recognize speaker emotional state on the basis of voice characteristics date back to the 1980s [77]. The initial motive for this research direction was the adaptation of an ASR system to emotionally stressed speech [78], but another motive appeared with the development of spoken dialogue systems, where it was useful to modify the dialogue strategy based on, e.g., user annoyance [79]. There are a number of emotions that can be easily represented in the activation-evaluation space [80], but classification of such a large number of emotions is difficult. Hence, classification space has been reduced to neutral and 6 archetypal emotions: anger, disgust, fear, joy, sadness, and surprise, which are the most obvious and distinct emotions [80]. It should be noted that archetypal emotions are not primary emotions in so-called “pallet theory,” where each emotion can be represented as a combination of the primary ones.

One of the important steps in the design of a speech emotion recognition system is the extraction of features that efficiently discriminate between emotions independently of lexical content, speaker, and acoustic environment. It is well known that prosodic features are correlated with emotions [80], which is why standard features used in emotion recognition systems include pitch, energy, and phone duration [81]. These features are also related to the voice quality that is related to the emotions [82]. Emotions affect speech energy distribution across a wide range of frequencies, thus spectral features such as MFCCs, linear prediction cepstral coefficients, log frequency power coefficients, and formants were further proposed [83, 84]. Feature extraction procedure starts with the segmentation of the input signals into 20–30 ms long frames shifted by 10 ms, since speech is a nonstationary signal. After that, the features extracted from a chosen segment (corresponding to a particular phoneme, syllable, word, or sentence) are mapped into a single vector using functionals such as mean, second moment, contour slope, and range. Hereafter, features “condensed” in such a way represent the input of standard classification algorithms such as linear Bayes [85], *k* nearest neighbours [85, 86], support vector machines [87], GMM [86], and artificial neural networks [88]. On the other hand, such frame-based features can also be classified as a sequence using HMM [84] and RNN [89]. Besides low-level acoustic features, individual words or sequences of words obtained by ASR can also be used to perform emotion classification [90]. After a huge success of convolutional neural networks (CNN) in image classification, where lower layers of the network perform feature extraction, some research groups have tried to implement CNN in the same manner to obtain features [91, 92]. Since speech emotion recognition is a scarce data problem, one of the future trends will be the application of semisupervised learning [93]. More details about features, classification algorithms, and databases can be found in [94–97].

### 3.3. Progress in the Development of Text-to-Speech Synthesis

The very first “speech machines” were mechanical devices capable of producing single phonemes, and some of their combinations were introduced by Christian Kratzenstein and Wolfgang von Kempelen at the end of eighteenth century [98]. The VODER, presented in 1939 by Homer Dudley, can be considered as the first synthesizer which could generate whole sentences [99]. The first full TTS system for English was introduced in 1968 by Teranishi and Umeda [100]. It was an articulatory-based system which could perform text analysis and determine pauses in text using a sophisticated parser [101].

However, it was not until concatenative synthesizers were invented, that TTS gained widespread usage. The idea of concatenative TTS is to concatenate appropriate parts of a prerecorded database [102]. If the goal is domain-specific synthesis or a very large speech database is available, this approach can produce high-quality speech. However, there are audible glitches at the concatenation points if the appropriate units cannot be found in the database. The method is also extremely inflexible in terms of changing the speaking style or the voice of the speaker; it requires a whole new database to be recorded and annotated.

As applications of TTS became more popular and more widely used, the necessity of algorithms that could produce different voices and speaking styles from smaller databases has grown. From around 2000, statistical parametric speech synthesis, where the spectrum, fundamental frequency, and duration of speech were modelled by multispace probability distribution HMMs and multidimensional Gaussian distributions [103], became popular. The HMM synthesis enables transformation of speaker-independent system toward a target speaker using a very small amount of speech data [104], creating expressive voices [105], as well as multilingual voices [106]. However, this method never achieved the naturalness of concatenative TTS. One of the main problems is the signal smoothness caused by modelling similar contexts with the same Gaussian mixtures. Another big problem introduced with parametric methods is the usage of vocoder, a system that produces speech waveforms from predicted acoustic features. Vocoders, although significantly improved over the time, introduce some artefacts which affect the overall quality of generated speech. A detailed review of HMM-based speech synthesis can be found in [107].

The first attempts to use neural networks in speech synthesis can be found in [108]. However, the recent development of hardware, especially graphical processing units (GPUs), has popularized this approach and established its dominant status in the TTS research society. Deep neural networks (DNN) replaced decision trees and Gaussian mixture models in mapping input linguistic features to output acoustic features, enabling nonlinear mappings [109]. Although simple feedforward NN with several hidden layers and sigmoid or tangent hyperbolic activations are sufficient for the production of intelligible and natural sounding synthetic speech, introduction of LSTM (long short-term memory) units has brought further improvement into the quality of synthesized speech [110]. Some improvements were also reported by introducing generative adversarial networks [111] and stacked bottleneck features [112].

DNNs have not only just enabled generating synthetic speech of high quality but also introduced many possibilities for production of speech in different voices and speaking styles. A majority of methods for creating new DNN voice using limited amount of training data is based on usage of multispeaker models. In multispeaker modelling, a large database consisting of multiple speakers is required. Each speaker is usually represented with less data than in case of single-speaker modelling. Due to a variety of contextual information and better network generalization, the quality of speech produced with multispeaker models is similar or even better than speech obtained with single speaker models. Speaker identity in multispeaker systems can be represented in several ways. One group of approaches is based on the use of a unique vector for each speaker. This vector can be represented as *i* vector [113] or just one-hot vector [54] and is used in training as extension of standard input or additional input to any of the hidden layers. Another group of methods for representing speaker identity is based on splitting network to speaker-specific and shared parts. In [53], separate output layers for each speaker have been introduced. In [114], even language-dependent parts of the network have been added, but this approach requires data from the same speaker in multiple languages. Creating a new voice, whose samples have not been seen in the training phase, in a multispeaker framework is based on adapting only the speaker-dependent part of the network [53], estimating the speaker-specific vector for the new speaker [55] or adjusting the parameters of neurons in starting models [113]. As opposed to the usage of multispeaker models as starting models for adaptation, in [115], adaptation starting from a single speaker model is investigated. It has been shown that only ∼10 min of target speaker voice is required in order to produce synthetic speech in target speaker's voice reaching the quality of conventional methods built on several hours of speech database. The hypothesis was that the models of speakers A and B are more similar than a randomly initialized model and the model of speaker B, consequently requiring less data to train the model of speaker B starting from the model of speaker A than starting from a randomly initialized model.

Synthetic speech should convey not only just information but also paralinguistic information such as emotional state. There is also a need to support some task-specific speech styles such as news, commercials, storytelling, and warnings [116]. It has been shown that emotion, mood, and sentiment affect attention, memory, performance, judgement, and decision-making in humans [117], which supports the necessity of using different speaking styles in synthetic speech for many applications. Three different methods for style modelling are compared in [118]. The presented methods are based on ideas introduced in multispeaker modelling using input codes, network adaptation, and separated output layers. It has been shown that only ∼5 min of speech per style is sufficient in order to produce speech of acceptable quality in a specific style. Using input codes for representing different styles is also presented in [119, 120]. There have also been attempts at style transplantation, i.e., producing speech in the voice of speaker A in style X without having any sentence from speaker A in style X in the training data, in which case the network is forced to learn the style X from other speakers in the training database [121, 122].

Although DNNs have shown to be extremely powerful and flexible, for a long time, one of their main disadvantages in speech synthesis has been their dependence on the usage of a vocoder. For the first time in 2016, raw audio samples were directly predicted by DNN using WaveNet architecture [51]. This model is fully probabilistic and autoregressive, with the predictive distribution for each audio sample conditioned on all previous ones. When conditioned on linguistic features derived from text and speaker identity, it can be used as TTS and it significantly outperforms all other TTS systems. The main drawbacks of this system are its need for extremely large databases and extreme computational power, although the synthesis has since been accelerated by the introduction of approaches such as Parallel WaveNet [123]. A similar model called DeepVoice was introduced in 2017 [124]. In DeepVoice, every part of TTS pipeline is replaced by a corresponding DNN. Its main drawback is the fact that all components of TTS system are trained independently, and it leads to a cumulative error in synthesized speech in the end.

As opposed to WaveNet and DeepVoice systems, which use lexical features as inputs, there are systems which use raw orthographic text as input, such as Tacotron [52], Tacotron 2 [125], and Deep Voice 3 [126]. Tacotron outputs spectrograms that are transformed to speech samples using Griffin–Lim algorithm, which also introduces artefacts in generated speech. On the other hand, the Tacotron 2 system-generated spectrograms are used for conditioning standard WaveNet architecture, which generates speech samples. DeepVoice 3 architecture can output spectrograms or other features which can be used as input to some waveform synthesis models. Adaptation to new speakers has also been investigated in end-to-end systems [127, 128] as well as synthesis in different styles [129, 130].

The main advantage of an integrated end-to-end TTS system is that requires minimal human effort since there is no need to label input data. Since in end-to-end systems, direct sample values are often predicted [29], the usage of 16 bit samples would make the prediction complicated and some type of quantization is performed. For this reason, improved coding and compression algorithms are important for TTS.

### 3.4. Dialogue Systems

Automatic speech recognition and speech synthesis are technologies with a long history. During the last five decades, a wide spectrum of algorithms shaped our knowledge within the speech technology field. With the recent advances in the world of deep learning and artificial neural networks, we are able to imitate to some extent the human auditory system sensitivity, recognition accuracy, human voice intelligibility and naturalness, emotions in synthesized speech, etc. As the result, machines are able to identify particular speakers, recognize human words in a noisy environment or to perform large-vocabulary continuous speech recognition with high accuracy. Furthermore, with a small amount of speech data from target speakers, they synthesize high quality speech, good enough to become a threat to automatic speaker verification systems.

One question that naturally arises is whether this is enough to achieve intelligent-like, natural and long-term human-machine speech interaction. Unfortunately, it is not.

Speech recognition and synthesis are only two of the six modules in a typical architecture of a speech dialogue system, depicted in Figure 4, and each of the six modules corresponds to certain cognitive aspects underlying the human language processing system. As a result, dialogue management becomes a complex structure that involves cooperation of several, quite different functional units. Leaving aside the division of dialogue systems into task-oriented and non-task-oriented, we must emphasize that the biggest challenge scientists face during the research in this area is to emulate human ability to understand meaning and conduct a conversation that is forward-looking, informative, and coherent. Regarding the dialogue initiatives, machines are successful in handling conversations that are system initiative (or single initiative). In such cases, the system completely controls the conversation and maintains the processes of speech recognition, meaning extraction, answer generation, and speech production. However, as it is known, natural dialogue is not deterministic and real improvements in human-machine speech interaction can be achieved only if adaptive behaviour with respect to the intention, the current context, and history of interaction are provided.

As a traditional paradigm shift, recent works in this area have addressed a series of data-driven, end-to-end trainable, non-goal-driven systems based on generative probabilistic models [131]. As such, these models can be viewed as artificial cognitive systems, aimed at grouping and carrying out traditional dialogue management tasks: language understanding, reasoning, decision-making, and natural language generation. They are corpus-based, data-driven dialogue systems, based on machine learning algorithms using corpora created from real word data. The statistics observed in dialogue corpora is the main knowledge for the optimization of parameters and variables.

It is worth pointing out that, besides the importance of domain knowledge, linguistic context has the crucial impact for active and engaging conversation. However, one of the main drawbacks of these approaches is related to sparsity issues that can be expected during integration of contextual information into statistical models. In the work of Sordoni et al. [132], the neural network architecture is used to address this problem, allowing the system to take into account the previous dialogue utterances. While modelling contextual information, the authors identify models for three linguistic entities in a conversation: the context (*c*), the message (*m*), and response (*r*). On that basis, they suggest three context-based generation models to estimate a response *r*=*r*_1_,…, *r*_T_ as follows:

(1) $$\begin{array}{c} p\left(r\;|\;c,m\right)=\;\overset{\text{T}}{\underset{t=1}{\prod }}p\left(r_{t}\;|\;r_{1},\ldots ,r_{t-1},c,m\right). \end{array}$$

This work extends the recurrent neural network language models (RNNLM) as a generative model of sentences [133]. As the basic principle in this neural network model, input vector, representing the current word at time instant *t*, is concatenated to the output from neurons in network context layer at time *t* − 1. In order to capture long-span dependencies together with semantic and syntactic similarities, the authors select word embedding as a continuous representation of words and phrases. Similar approaches already advanced classical language modelling, based on traditional *n*-gram language models [134, 135].

In line with this, many researches are trying to take advantage of combining neural network and end-to-end training possibilities with the large amount of freely available text or audio material from social media, movie scripts, etc. [136]. Serban et al. [137], for example, demonstrated introduction of latent variables to hierarchical recurrent encoder-decoder architecture. The research presented in [138] extended the hierarchical structure with the attention mechanism (word level attention and utterance level attention), taking into account that words and utterances in the context are differentially important.

Although end-to-end, statistical models have drawn most of the recent research on dialogue systems, many problems remain unresolved [131]. Neural network-based models are capable of handling large amount of data, but still it is hard to design an intelligent system based on imitating responses (especially if we take into account that the dialogue data for a specific domain are quite limited). Hence, to reduce these limitations, Mišković et al. [36] proposed a different, representational approach. This work extends the focus tree model, a cognitively inspired computational model of working memory that allows for adaptive dialogue management in human-machine interaction. The research not only is focused on improvements of speech recognition module but also points to possible new architectural aspect of dialogue systems. Following the assumption of the hierarchical and associative nature of human memory system and facts that the processing of the user's dialogue acts in human-machine interaction is always context-dependent, this model enables, to some extent, understanding of language and real word data.

## 4. Progress in Speech Signal Compression, Coding, and Transmission

**Figure 5:**
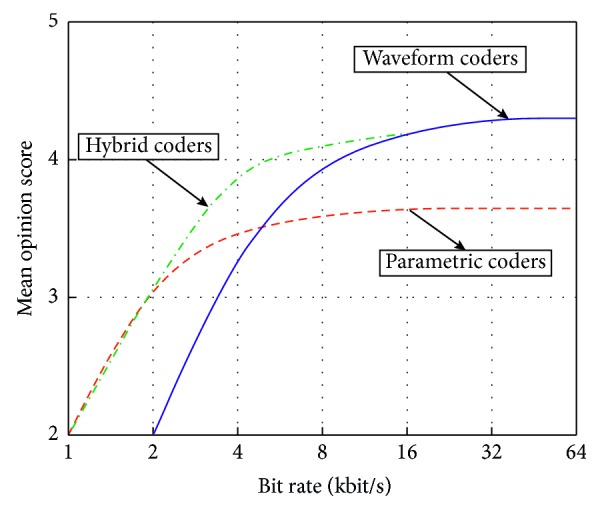
Speech signal quality according to MOS versus bit rate for various speech signal coding techniques.

In general, speech coders can be classified into three major categories depending on the applied coding technique: waveform coders, parametric coders, and hybrid coders. The primary idea behind a waveform coder design is to preserve the shape of a speech signal waveform, thus encoding information about the original time-domain waveform [4–6, 14, 139]. Such coders are broadly used in embedded applications due to several reasons: low cost of manufacturing, low computational resource usage, and high speech quality [4–7, 14, 139]. The simplest and most well-known type of waveform coders is pulse code modulation (PCM) coder, which is considered as a standard in digital telephony. One of the key advantages of PCM coders is that they are instantaneous, implying a coding delay of no more than one sample period [4]. Unlike waveform coders, which tend to reconstruct the original shape of the speech signal in time-domain, parametric coders reconstruct the speech signal from certain parameters that model the source signal, making no attempt to preserve the original shape of the waveform [4–7, 14, 139]. Due to this limitation, parametric coders are more signal dependent and less versatile. Additionally, compared to waveform coders, they provide a lower quality of speech signals. In parametric coders, human speech production mechanism is modelled with a time-varying filter, having coefficients commonly determined by the linear prediction analysis procedure. In the end, hybrid coders represent a class of coders, which combine features of both previously described classes of coders, namely, hybrid coders tend to preserve the shape of the signal in time domain and also exploit perceptive characteristics, that is, parametric approach [4–6, 14, 140]. Performance comparison of these three classes of coders is presented in Figure 5, where mean opinion score (MOS) is used as one of the standard subjective measures of reconstructed speech signal quality [4].

From Figure 5, one can conclude that waveform coders provide excellent quality of reconstructed speech signal and that they represent the best choice at bit rates higher than 16 kbits/s, whereas parametric coders cannot provide high quality regardless of the bit rate. On the other hand, parametric coders provide much better quality than waveform coders at low bit rates. Finally, hybrid coders are most suitable at medium bit rates. As for the purposes of speech synthesis and automatic speech recognition, the highest possible quality of reconstructed signal is desirable and waveform coders are usually considered as an adequate choice. Thus, what follows is focused on PCM and adaptive PCM (ADPCM) coding techniques.

### 4.1. Adaptive PCM

Speech signal can be considered as a nonstationary process, whose average power significantly fluctuates in time domain, resulting in a wide dynamic range [4]. However, speech signal can be considered as almost stationary in a short period of time (up to 30 ms). This means that speech signal has a highly predictable characteristics during short periods of time, which is suitable for utilizing adaptive quantization [4, 8–10, 141–147]. Commonly, adaptive quantization is frame-based, where frames are formed by dividing an input speech signal into sets of samples.

There are two fundamentally distinct categories of adaptive quantization techniques: forward and backward adaptive quantization techniques [141]. Forward adaptive techniques require transmission of additional information about the estimated gain, which is used for adaptation. Moreover, forward adaptive techniques require a longer processing delay than backward adaptive techniques as samples within a frame have to be stored in a buffer, in order to estimate predictable characteristics of every frame. When the gain is estimated and the quantizer is adapted, samples can be quantized and further transmitted to the decoder along with the quantized gain.

**Figure 6:**
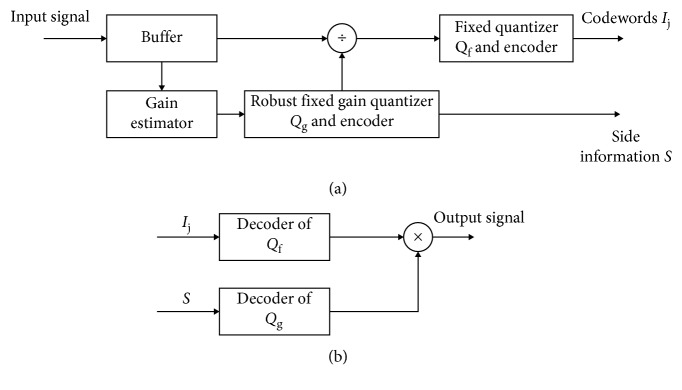
Forward adaptive PCM: (a) encoder; (b) decoder.

A general forward adaptive PCM model is presented in Figure 6 [8, 10]. The encoder is formed of two parts: a fixed (nonadaptive) part, consisting of a fixed quantizer *Q*_f_, and an adaptive part, consisting of a buffer, a gain estimator, one divider, and a fixed gain quantizer *Q*_g_. If *Q*_f_ is a piecewise linear *μ*-law quantizer designed for 8 bit/sample and *μ* = 255, the general forward adaptation model becomes a forward adaptive PCM model defined by G.711 standard [148].

Unlike forward adaptation, backward adaptation does not estimate characteristics of samples in a frame while encoding, which means that there is not additional information that has to be transmitted [149]. In fact, gain estimation is performed at the receiver after decoding, considering previously quantized samples. The simplest backward adaptive quantization model is based on uniform quantization with one codeword memory exploited for gain estimation and it is commonly referred as Jayant's model [4].

Advanced backward adaptive models commonly incorporate more sophisticated gain estimation methods, or variance $\left({\hat{\sigma }}_{y}^{2}\left(n\right)\right)$ estimation methods, which, for quantization of a current sample *x*(*n*), typically exploit a larger number of previously decoded samples *y*(*n* − *i*) [4]:

(2) $$\begin{array}{c} {\hat{\sigma }}_{y}^{2}\left(n\right)=\frac{1-\alpha }{1+\alpha }\overset{+\infty }{\underset{i=1}{\sum }}\alpha^{i-1}y^{2}\left(n-i\right), \end{array}$$

where *α* is a weighting parameter, which can take values 0 < *α* < 1. Parameter *α* defines a learning period, that is, a time required for variance estimation [4]:

(3) $$\begin{array}{c} L=\frac{1+\alpha }{1-\alpha }. \end{array}$$

**Figure 7:**
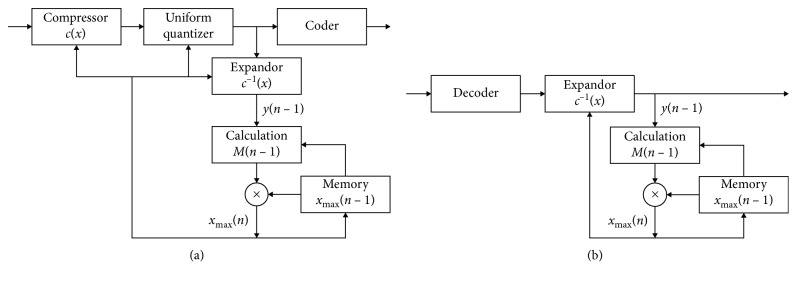
One of the realizations of backward adaptive PCM with one codeword memory: (a) encoder; (b) decoder.

Equation (2) can be written in the following recursive form:

(4) $$\begin{array}{c} {\hat{\sigma }}_{y}^{2}\left(n\right)=\frac{1-\alpha }{1+\alpha }y^{2}\left(n-1\right)+\alpha {\hat{\sigma }}_{y}^{2}\left(n-1\right), \end{array}$$

which is straightforwardly used in the simplest mathematical model of Jayant's backward quantizer with one codeword memory. One of the realizations of backward adaptive PCM with one codeword memory that incorporates a widely used companding quantization model is shown in Figure 7, where *M*(*n* − 1) denotes a step size multiplier, used for adaptation, and *c*(*x*) and *c*^−1^(*x*) are a compressor function and an expandor function, respectively.

### 4.2. Dual-Mode Quantization

**Figure 8:**
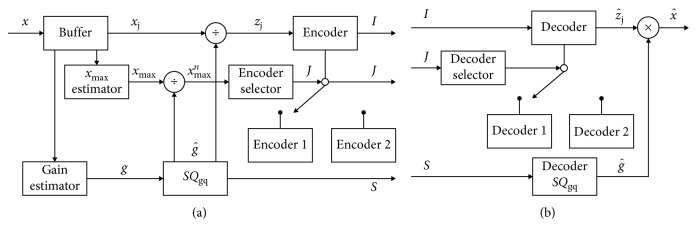
Dual mode quantization scheme: (a) encoder; (b) decoder.

Dual-mode and adaptive dual-mode quantizers belong to a relatively new class of quantizers whose design is based on multiparameter adaptation, such as variance and maximum amplitude [8, 9, 11]. Depending on their purpose and application, they can perform quantizer adaptation according to the frame variance and to the frame maximum amplitude *x*_max_ and also according to the subframe maximum amplitude. By utilizing two quantizers, which compose the dual-mode system, and by applying switched technique, it is possible to achieve a better quality of the quantized signal, or a higher compression, compared to the common single-mode quantizers. In Figure 8, a dual-mode quantization scheme is shown, where Encoder 1 and Decoder 1 are related to the quantizer applied for processing signals having restricted amplitude range, whereas Encoder 2 and Decoder 2 are related to the quantizer applied for processing the signals having unrestricted amplitude range [8, 9, 11]. The switched process is frame-based, and it is performed so that the restricted quantizer is used in the case if all samples within a frame belong to the restricted quantizer's support region, while the unrestricted quantizer is used otherwise [8, 9, 11]. The main idea behind such quantization model is to enable a more preferable selection of the restricted quantizer, with a narrower support region, than the unrestricted one, since, in such a manner, an increase of the signal to quantization noise ratio can be provided.

Considering that speech signal can be described using Gaussian probability density function (PDF) or Laplacian PDF, which is heavy-tailed, it is expected that only small percentage of speech frames will have some samples of large values. However, this also depends on the size of a frame. Consequently, the support region threshold values for both quantizers should be chosen so that the restricted quantizer usage should be dominant, but taking also into account the frame size and the whole input signal dynamics in order to achieve a minimum of the total distortion introduced in the quantization process [8, 9, 11].

### 4.3. Differential Pulse Code Modulation

Differential pulse code modulation (DPCM) represents a simple but high-quality speech signal coding scheme for middle bit rates. It initially exploited uniform quantization and the first-order prediction [150, 151]. As it was already discussed, speech signal has highly predictable characteristics within a frame, which is exploited to reduce the dynamic range of amplitudes for quantization in the DPCM scheme [4, 12, 13, 152, 153]. In particular, DPCM predicts the next sample amplitude value and encodes the difference between the predicted value and the value of the current input signal amplitude. Due to the high correlation, these differences have much smaller values compared to ones of the input signal samples, so that the dynamic range of amplitudes is significantly reduced before quantization. Accordingly, with a suitable design of a DPCM system, a certain distortion may be provided at lower bit rates compared to the PCM system. In other words, a worthy compression may be achieved with the DPCM system compared to the PCM.

**Figure 9:**
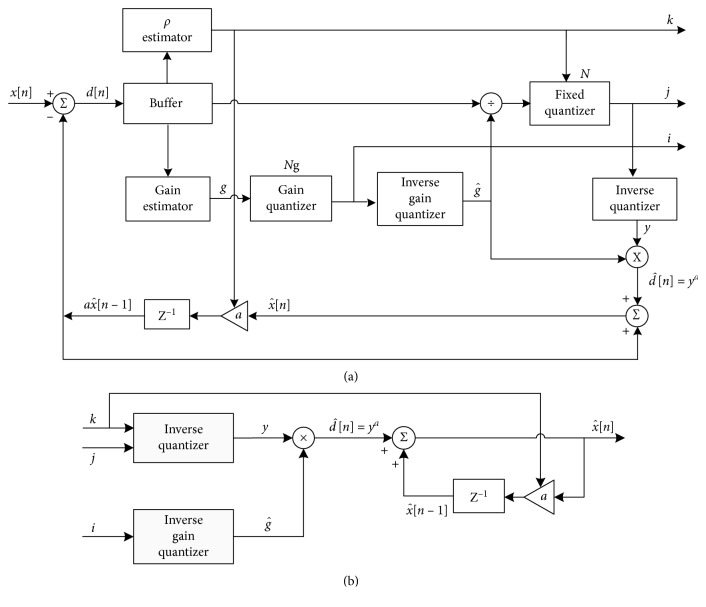
DPCM: (a) encoder; (b) decoder.

More sophisticated solutions may incorporate prediction of a higher order or other kinds of quantization models, such as a gain-adaptive quantization model [154].  Figure 9 shows a DPCM scheme with incorporated simple first-order predictor and forward gain-adaptive quantizer based on optimal companding model [151].

In the DPCM system given in Figure 9, the reconstructed speech signal $\hat{x}$ is determined by

(5) $$\begin{array}{c} \hat{x}\left[n\right]=a\cdot \hat{x}\left[n-1\right]+y^{a}\left[n\right], \end{array}$$

where *y*^*a*^ denotes the output of the adaptive quantizer, whereas *n* denotes the *n*-th sample of the signal. It can be noted that the value of parameter *a* depends on the nature of the input signal. If an input signal is highly correlated, it is preferred to use values close to 1 (e.g., *a* = 0.8), whereas values close to zero are preferred for lowly correlated signals (e.g., *a* = 0.3). However, the choice of parameter *a* is not an easy task even if adaptation is applied. The determination of linear predictor coefficients can be done using methods that are based on statistical learning such as least mean squares (LMS) estimation method [155]. LMS search algorithm reduces distortion by adapting coefficients for each input sample, and its main features, which attract researches, are low computational complexity, proof of convergence in stationary environment, unbiased convergence in the mean to the Wiener solution, and stable behaviour when implemented with finite-precision arithmetic [156]. Moreover, coefficients of linear predictor as well as determination of other important parameters for quantizer design may be determined by exploiting artificial neural networks or regression methods.

## 5. Conclusions

This review article has provided an overview in the recent development of speech technologies and other scientific areas related to them, mostly due to the development of the new machine learning paradigm, which has had a tremendous impact in this domain. Apart from natural speech production and speech perception, understanding of cognitive aspects of speech communication is very important for future HCI systems including both spoken language understanding and generation as language technologies. The machine learning paradigm has had a great impact on automatic speech recognition (ASR) and text-to-speech synthesis (TTS) as basic speech technologies. It is expected that ASR systems based on deep learning and adaptive algorithms in the near future will be able to recognize spontaneous speech in complex acoustic environments, with the accuracy that will surpass the corresponding ability of humans. Synthetic speech has already reached such quality that is hard or impossible to differentiate from human speech. With flexibility of changing speaker and style, HCI is becoming as pleasant and natural as human-human interaction. Unsupervised and reinforcement-based machine learning algorithms will also develop further, which will, in turn, bring about progress in areas where large data sets are not available, as is the case in speech analysis for speech recognition and synthesis for under-resourced languages. A short overview of speech coding techniques and of current progress in adaptive scalar quantization has been presented as the quality of digitized and compressed speech signal is important for accurate automatic speech signal detection and synthesis. Although these techniques can be designed to be robust in a wide dynamic range of speech signal variations, or to be frame-adaptive, one can anticipate that machine learning tools of increasing popularity will lead to novel solutions, which will improve performances of various systems by adapting predictive coefficients. To conclude, we are witnessing an increasingly fast progress in the field of speech signal processing due to machine learning paradigms, and it appears very hard to predict what they will bring about next and how soon that can be expected.
